# Reduced *in vivo* hepatic proteome replacement rates but not cell proliferation rates predict maximum lifespan extension in mice

**DOI:** 10.1111/acel.12414

**Published:** 2015-11-06

**Authors:** Airlia C. S. Thompson, Matthew D. Bruss, John C. Price, Cyrus F. Khambatta, William E. Holmes, Marc Colangelo, Marcy Dalidd, Lindsay S. Roberts, Clinton M. Astle, David E. Harrison, Marc K. Hellerstein

**Affiliations:** ^1^ Department of Nutritional Science and ToxicologyUniversity of California at BerkeleyBerkeleyCA94720USA; ^2^ KineMed Inc.EmeryvilleCA94608USA; ^3^ The Jackson LaboratoryBar HarborME04609USA; ^4^ Department of BiologyStanford UniversityStanfordCA94305USA; ^5^ PPD Inc.MiddletonWI53562USA; ^6^ Department of Chemistry and BiochemistryBrigham Young UniversityProvoUT84602USA; ^7^ KineMed Inc.EmeryvilleCA94608USA

**Keywords:** calorie restriction, cell proliferation, maximum lifespan extension, proteome dynamics, rapamycin, Snell Dwarf

## Abstract

Combating the social and economic consequences of a growing elderly population will require the identification of interventions that slow the development of age‐related diseases. Preserved cellular homeostasis and delayed aging have been previously linked to reduced cell proliferation and protein synthesis rates. To determine whether changes in these processes may contribute to or predict delayed aging in mammals, we measured cell proliferation rates and the synthesis and replacement rates (RRs) of over a hundred hepatic proteins *in vivo* in three different mouse models of extended maximum lifespan (maxLS): Snell Dwarf, calorie‐restricted (CR), and rapamycin (Rapa)‐treated mice. Cell proliferation rates were not consistently reduced across the models. In contrast, reduced hepatic protein RRs (longer half‐lives) were observed in all three models compared to controls. Intriguingly, the degree of mean hepatic protein RR reduction was significantly correlated with the degree of maxLS extension across the models and across different Rapa doses. Absolute rates of hepatic protein synthesis were reduced in Snell Dwarf and CR, but not Rapa‐treated mice. Hepatic chaperone levels were unchanged or reduced and glutathione S‐transferase synthesis was preserved or increased in all three models, suggesting a reduced demand for protein renewal, possibly due to reduced levels of unfolded or damaged proteins. These data demonstrate that maxLS extension in mammals is associated with improved hepatic proteome homeostasis, as reflected by a reduced demand for protein renewal, and that reduced hepatic protein RRs hold promise as an early biomarker and potential target for interventions that delay aging in mammals.

## Introduction

Age is a risk factor for numerous diseases including cancer, diabetes, and neurodegenerative diseases (Miller, 2002). The potential social and economic consequences of the predicted increase in the proportion of the US population that is 65 years and over (West *et al*., 2014) have sparked great interest in the development of interventions that extend healthspan, the time in which an individual remains active and in good health. The extension of maximum lifespan (maxLS, generally defined as the age at which 90% of individuals in a given population have died) has proven to be a useful outcome metric, as it in principle represents a general slowing of the aging process rather than the curing of one specific age‐related disease.

Over the last 50 years, several dietary, genetic, and pharmacological interventions have been identified that extend maxLS in laboratory animals, including mice (Weindruch *et al*., 1986; Liang *et al*., 2003; Miller *et al*., 2005; Harrison *et al*., 2009, 2014). Understanding the molecular underpinnings of the aging process and the biochemical pathways affected by interventions that attenuate the development of age‐related diseases is a high priority. In particular, identifying metrics of biological processes, or biomarkers (BMs), that are involved in the slowing of aging in laboratory mammals will be essential for guiding the future development of interventions to extend human healthspan.

To identify such processes that play an etiologic role in age‐related disease, our approach has been to test potential flux‐based BMs of maxLS extension. This is an approach our group has previously applied to identify causal pathway BMs in other conditions, such as neurodegeneration (Fanara *et al*., 2012), fibrotic disease (Decaris *et al*., 2015), and cancer (Messmer *et al*., 2005). The concept underlying this strategy is that the activity of a metabolic process is best characterized by the molecular flux rate traversing the pathway and that changes in the flux rates of metabolic processes that play a causal role in the functional alterations of the condition may manifest earlier and more sensitively than static pathologic changes or complex clinical outcomes. Optimally, the functional role and measurement technique for these molecular processes will be translatable into human studies.

This rate‐based BM approach may be particularly promising in combination with what is currently the most robust program for testing proposed interventions for extension of maxLS in mammals: the National Institute on Aging's (NIA) Interventions Testing Program (ITP; Miller *et al*. 2007). These studies are the current gold standard for evaluating changes in lifespan (LS) in genetically heterogeneous mice but are time‐ and resource‐intensive, limiting the number of interventions that can be tested each year. An initial screening strategy based on a panel of early BMs of maxLS extension could be used to further refine which candidate interventions should be prioritized for inclusion into LS studies in mice. This represents an attractive approach for identifying interventions with the potential to extend human healthspan.

No previous studies have identified robust and early BMs of maxLS extension applicable across diverse genetic backgrounds and interventions. Here, we used stable‐isotope mass spectrometric measurement tools to screen for BMs based on the activity of targeted physiologic pathways believed to be involved in the aging process. These measurements were performed in three different yet well‐established mouse models of maxLS extension, each on a different genetic background and each at relatively early time points in the LS of the model. The three models evaluated were Snell Dwarf mice, which are homozygous for a loss‐of‐function mutation in the Pit1 gene involved in anterior pituitary development; calorie‐restricted (CR) mice, in which calories are reduced without malnutrition; and mice treated with rapamycin (Rapa), a macrolide that inhibits the serine/threonine kinase mechanistic target of rapamycin (mTOR; Blackwell *et al*., 1995; Flurkey *et al*., 2002; Harrison *et al*., 2009; Miller *et al*., 2011, 2013). Our broad objective was to identify early rate‐based BMs of maxLS extension that meet several criteria, including that they (i) manifest soon after the initiation of an intervention; (ii) are predictive of maxLS extension at early time point measurements; (iii) are not sex‐, strain‐ or intervention‐specific, and (iv) reveal the activity (i.e., flux) of targeted biological pathways. In the studies presented here, we evaluated two candidate BMs of maxLS extension: reduced *in vivo* cell proliferation rates across multiple cell types and reduced *in vivo* proteome turnover rates.

A reduction in cell proliferation rates has been hypothesized to contribute to maxLS extension by inhibiting the promotional phase of carcinogenesis and delaying cellular replicative senescence (de Magalhães & Faragher, 2008). Consistent with this hypothesis, CR in rodents results in dramatic reductions in global cell proliferation rates as well as the preservation of the proliferative capacity of many cell types later in life (Lok *et al*., 1990; Wolf *et al*., 1995; Bruss *et al*., 2011). In contrast, premature aging and shortened maxLS are associated with early‐life hyperproliferation and premature loss of the proliferative capacity of cells in both mice and humans (Pendergrass *et al*., 1993; Bridger & Kill, 2004).

A reduction in protein synthesis rates or slowing of protein replacement rates (RRs) (turnover) might in principle reflect preserved proteome homeostasis (proteostasis), which normally declines with age (Rattan, 2010). Proteostasis is achieved through the proper coordination of the synthesis, folding, transport, modification, and degradation of proteins. The maintenance of proteostasis requires translational fidelity, molecular chaperones to facilitate protein folding and the efficient detection, repair and, if necessary, replacement of misfolded and/or damaged proteins that might otherwise compromise cellular function. A reduction in protein synthetic burden may preserve proteostasis by limiting the accumulation of misfolded and/or damaged proteins, possibly by increasing translational fidelity, chaperone capacity, and/or proteolytic capacity (Hipkiss, 2007; Kapahi *et al*., 2010; Conn & Qian, 2013; Sherman & Qian, 2013). In addition, when global protein synthesis is inhibited, differential translational upregulation of mRNAs encoding proteins involved in somatic maintenance and stress responses has been reported (Yamasaki & Anderson, 2008; Kapahi *et al*., 2010). Conversely, a primary reduction in damage to proteins, induction of chaperone synthesis, or a primary increase in proteolytic capacity (i.e., improved editing) could improve proteostasis independently of the absolute rate of protein synthesis in tissues.

Consistent with the model that alterations in protein synthesis and proteostasis play roles in LS determination, numerous models of LS extension in yeast, worms, flies, and mice are characterized by reduced signaling and/or expression of machinery involved in protein synthesis as well as enhanced expression of factors involved in somatic maintenance and stress responses (McElwee *et al*., 2007; Harrison *et al*., 2009; Kennedy & Kaeberlein, 2009). Conversely, impaired protein quality control results in features of premature aging and shortened LS in mice (Min *et al*., 2008).

Accordingly, the goal of the work presented here was to test the hypothesis that reduced cell proliferation rates, reduced protein synthesis rates, reduced protein RRs (prolonged half‐lives), or other proteome alterations are early BMs of maxLS extension in mice. In all three models, we measured the *in vivo* proliferation rates of epidermal, liver, and mammary cells, as well as the *in vivo* RRs, relative pool sizes (RPS), and within‐proteome absolute synthesis rates of over a hundred hepatic proteins across multiple gene ontologies.

## Results

### A reduction *in vivo* cell proliferation rates is not an early biomarker of maxLS extension in mice

To determine the extent to which a reduction in cell proliferation may be an early BM of maxLS extension in mice, we measured changes in the *in vivo* proliferation rates of epidermal, liver, and mammary cells in Snell Dwarf, CR, and Rapa‐treated mice. These cell types were chosen because they have previously revealed sensitive, dose‐dependent reductions in proliferation rates in response to CR regimens (Bruss *et al*., 2011 and data not shown).

**Figure 1:**
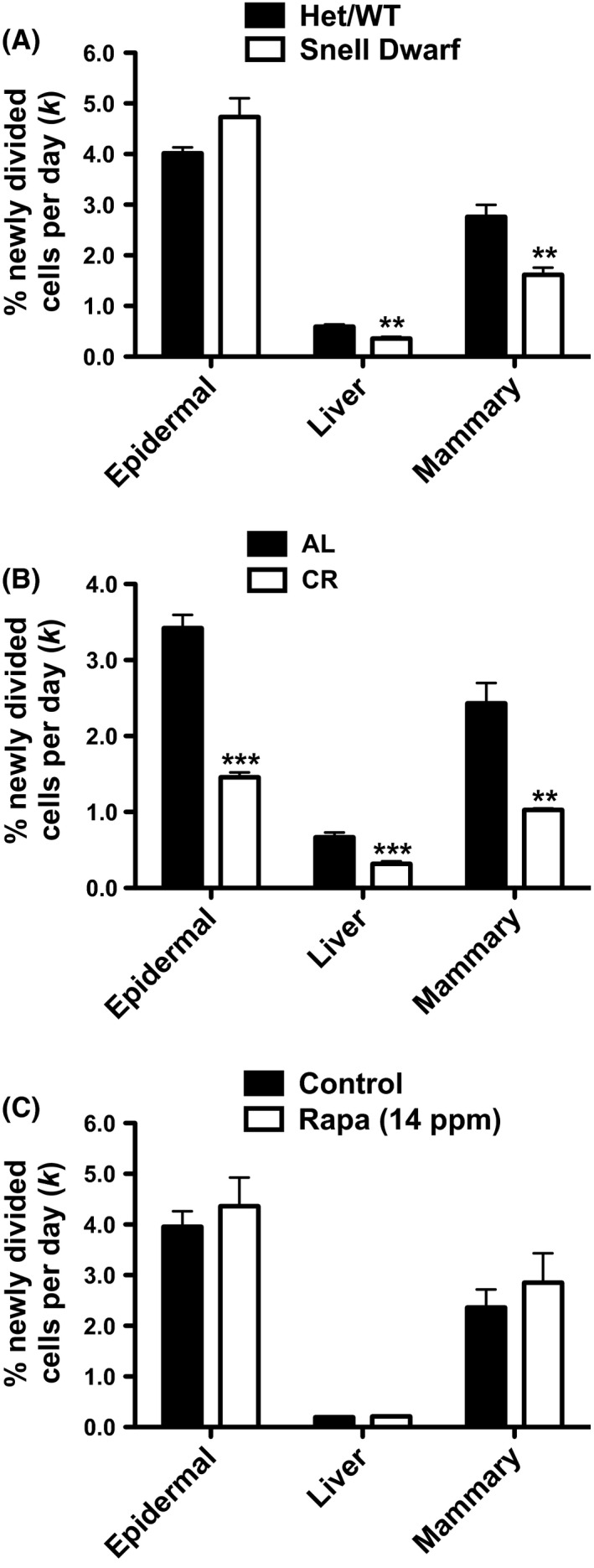
*In vivo* cell proliferation rates. Fractional replacement rates (*k*, expressed as % newly divided cells per day) of epidermal, liver, and mammary cells in A) Snell Het/WT vs. Dwarf (*n* = 5–6 per group), B) AL vs. CR (*n* = 5–6 per group), and C) control vs. Rapa (14 ppm) mice (*n* = 5–12 per group). Values are expressed as mean ± SEM. Student's unpaired two‐tailed *t*‐tests were used for all between‐group analyses (**P < 0.003, ***P < 0.0008).

In 6‐ to 7‐month‐old female Snell Het/WT and Dwarf DW/J mice, proliferation rates of liver and mammary cells were significantly reduced in Snell Dwarf compared to Het/WT mice (39.5% and 41.5% reduced, respectively). However, there was no difference in the proliferation rates of epidermal cells in Snell Dwarf compared to Het/WT mice (Fig. 1A).

In 4‐month‐old female C57BL/6 mice after 6 weeks on either an AL or 25% CR diet, proliferation rates of epidermal, liver, and mammary cells were significantly reduced in CR compared to AL mice (57.4%, 52.2%, and 57.7% reduced, respectively), consistent with previous reports (Fig. 1B; Lok *et al*., 1990; Bruss *et al*., 2011).

In 8‐month‐old female UM‐HET3 mice after 4 months on either a control diet or a diet containing 14 ppm Rapa [Rapa (14 ppm)], there were no significant differences in the proliferation rates of any of the cell types between groups (Fig. 1C). This lack of any significant effect of Rapa treatment on the proliferation rates of any of these cell types contrasts with reports that Rapa reduces the proliferation rates of HepG2 liver cells *in vitro* and muscle cells *in vivo* (Varma & Khandelwal, 2007; Drake *et al*., 2013). Divergence from these previously published results may be explained by differences in the dose and method of Rapa administration, differences in the *in vitro* versus the *in vivo* effects of Rapa, or differences in cell types. However, our observation that the *in vivo* proliferation rate of liver cells was unchanged is consistent with a previous study that used a very similar experimental design (Drake *et al*., 2013).

The data from these three models provide evidence that reductions in the *in vivo* proliferation rates of the three cell types analyzed, although associated with maxLS extension in some settings, are not consistent across all models tested and, therefore, are not ideally suited as early BMs of maxLS extension in mice. It remains to be determined whether the proliferation rates of other cell types may serve as such BMs.

### A reduction in *in vivo* hepatic proteome RRs may be an early biomarker of maxLS extension in mice

**Figure 2:**
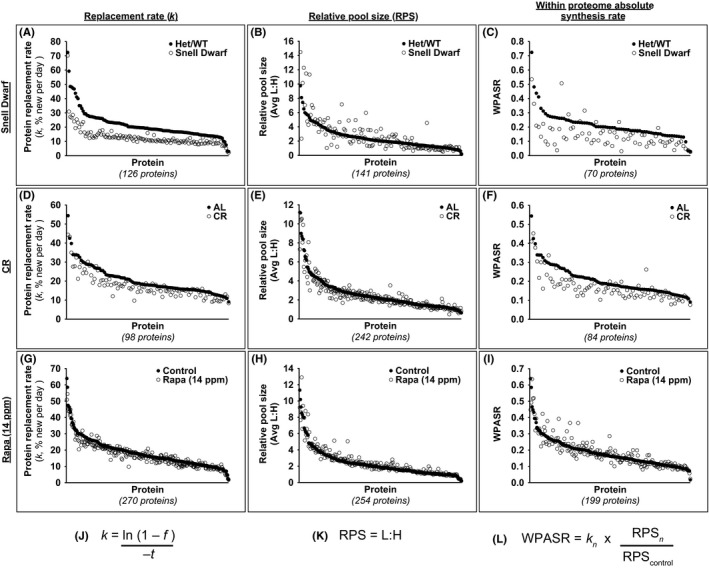
*In vivo* RR (*k*), RPS, and WPASR values for individual hepatic proteins. Protein RRs (*k,* expressed as % new per day), relative pool sizes (RPS), and within‐proteome absolute synthesis rates (WPASR) of hepatic proteins in A–C) Snell Het/WT vs. Dwarf, D–F) AL vs. CR, and G–I) control vs. Rapa (14 ppm) mice (*n* values per protein per parameter are provided in Spreadsheet S1). For each model, each vertical pair of solid and open symbols represents the mean RR (*k)*, RPS, or WPASR value for an individual protein in the control and experimental group, respectively. For clarity, protein names are not listed and proteins are sorted in descending order based on control group values. J) Equation used to calculate RR (*k*), where *f* is the fraction of protein newly synthesized during the labeling period (as measured by deuterium incorporation) and t is the number of days of heavy water labeling. K) Equation used to calculate RPS, where L is the abundance of the light version of a given protein coming from control or experimental animals and H is the abundance of the heavy version of that same protein coming from the internal heavy SILAM standard. L) Equation used to calculate WPASR, where *n* represents either the control or experimental group.

To determine the extent to which a broad, proteome‐wide reduction in protein synthesis or RRs (or other proteome alterations) may serve as early BMs of maxLS extension in mice, we measured *in vivo* hepatic proteome dynamics and relative protein concentrations in Snell Dwarf, CR, and Rapa‐treated mice. To capture changes in both the RR constants (*k*, fraction new protein per day) and RPS of individual proteins, we combined heavy water labeling with internal Stable Isotope Labeling in Mammals (SILAM) standards and measured both by LC‐MS/MS. The rate of incorporation of deuterium from heavy water into proteins was used to calculate RRs, as previously described (Price *et al*., 2012b). To calculate the RPS of proteins, liver homogenates were spiked with an exogenous heavy‐labeled SILAM standard (derived from the liver of a mouse fully labeled with ^15^N). The abundance of the light (L) version of a given protein represents the pool size in the study animal and was compared to the abundance of the heavy (H) version of that same protein derived from the SILAM standard. The L:H ratio therefore represents the RPS for individual proteins. Finally, the synthesis rate of a protein is the product of its pool size (concentration) times its RR (*k*). For those proteins for which both *k* and RPS data were available, we calculated within‐proteome absolute synthesis rates (WPASR) (see Fig. 2 for proteome metric equations). We term this ‘within proteome’ because the SILAM approach corrects for different ionization efficiencies and yields among peptides by comparing each to its cognate from the added ^15^N‐labeled standard, thereby revealing relative changes in concentration from sample to sample within the measured proteome, but does not reliably quantify true concentrations in the tissue (Wu *et al*., 2004).

#### Snell Dwarf model

**Figure 3:**
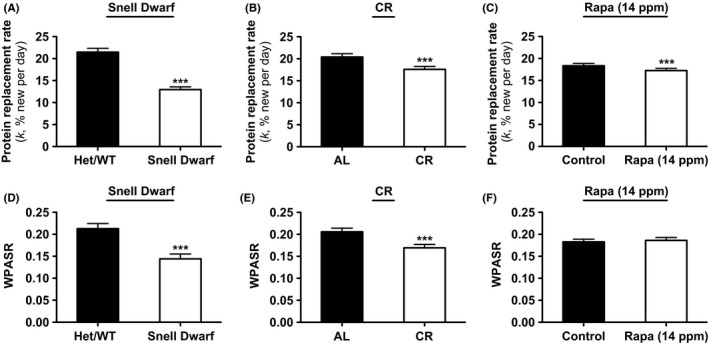
Mean *in vivo* hepatic proteome RR (*k)* and WPASR values. Hepatic protein RRs (*k*) in A) Snell Het/WT vs. Dwarf (*n* = 126 proteins), B) AL vs. CR (*n* = 98 proteins), and C) control vs. Rapa (14 ppm) mice (*n* = 270 proteins). Within‐proteome absolute synthesis rates (WPASR) of hepatic proteins in D) Snell Het/WT vs. Dwarf (*n* = 70 proteins), E) AL vs. CR (*n* = 84 proteins), and F) control vs. Rapa (14 ppm) mice (*n* = 199 proteins). Values are expressed as mean ± SEM. Paired two‐tailed heteroscedastic *t*‐tests were used for all between‐group analyses (***P < 0.0001). Rapa (14 ppm) data from Rapa study 1.

Hepatic proteomic analyses were conducted in 5.5‐ to 7‐month‐old female Snell Het/WT and Dwarf DW/J mice. We determined *k* values for 126 proteins, RPS values for 141 proteins, and WPASR values for 70 proteins in both Het/WT and Dwarf mice. Defining a change as plus or minus 10% of control value (Price *et al*., 2012b), *k* values were reduced for almost all (97.6%) of the identified proteins, while no proteins had increased *k* values in Snell Dwarf compared to Het/WT mice. RPS values were reduced for 35.5% and increased for 40.4% of the identified proteins in Snell Dwarf compared to Het/WT mice. Moreover, WPASR values were reduced for 86.0% and increased for only 10.0% of the identified proteins in Snell Dwarf compared to Het/WT mice (Fig. 2A–C, Table 1, and Spreadsheet S1). Both the mean hepatic proteome RR and mean WPASR were profoundly and significantly reduced in Snell Dwarf compared to Het/WT mice (mean ratio of Dwarf *k* to Het/WT *k*: 0.612; and mean ratio of Dwarf WPASR to Het/WT WPASR: 0.689; Fig. 3A,D and Table 1). These findings are consistent with reports of reduced translation initiation signaling and reduced rates of protein synthesis and replacement for mixed hepatic proteins, as measured by [4‐^3^H]‐phenylalanine incorporation, in Snell Dwarf compared to WT mice (Bates & Holder, 1988; Hsieh & Papaconstantinou, 2004).

**Table 1:** Summary of global *in vivo* hepatic proteome alterations

Model	Mean Experimental: control		# of proteins for which data were collected for parameter			# of proteins with ≥10% reduction in parameter (%)			# of proteins with ≥10% increase in parameter (%)		
	*k*	WPASR	*k*	RPS	WPASR	*k*	RPS	WPASR	*k*	RPS	WPASR
Snell Dwarf	0.612	0.689	126	141	70	123 (97.6%)	50 (35.5%)	60 (86.0%)	0 (0.0%)	57 (40.4%)	7 (10.0%)
CR	0.867	0.833	98	242	84	59 (60.2%)	92 (38.0%)	61 (72.6%)	1 (1.0%)	48 (19.8%)	4 (4.8%)
Rapa (14 ppm)	0.946	1.02	270	254	199	88 (32.6%)	26 (10.2%)	49 (24.6%)	28 (10.4%)	81 (31.9%)	55 (27.7%)

Experimental refers to the longer‐lived group within each model [Snell Dwarf, CR, or Rapa (14 ppm)], and control refers to the control group within each model (Snell Het/WT, AL, and control, respectively). *k *= protein replacement rate (expressed as % new protein per day). RPS, relative pool size; WPASR, within‐proteome absolute synthesis rate. Rapa (14 ppm) data from Rapa proteomics study 1.

#### CR model

Hepatic proteomic analyses were conducted in 18‐month‐old male C57BL/6 mice after 14 months on either an AL or 40% CR diet. Data from some of these AL and CR mice were reported previously (Price *et al*., 2012b); however, the data presented here include an additional heavy water labeling time point (2 days) as well as new SILAM analyses. We determined *k* values for 98 proteins, RPS values for 242 proteins, and WPASR values for 84 proteins in both AL and CR mice. Values of *k* were reduced for 60.2% and increased for only 1.0% of the identified proteins in CR compared to AL mice. RPS values were reduced for 38.0% and increased for 19.8% of the identified proteins in CR compared to AL mice. WPASR values were also reduced for 72.6% and increased for only 4.8% of the identified proteins in CR compared to AL mice (Fig. 2D–F, Table 1, and Spreadsheet S1). Both the mean hepatic proteome RR and mean WPASR were significantly reduced in response to CR (mean ratio of CR *k* to AL *k*: 0.867; and mean ratio of CR WPASR to AL WPASR: 0.833; Fig. 3B,E and Table 1). These results are consistent with a recent study employing short‐term (10 weeks) 40% CR in older mice (25‐month‐old). Using heavy‐leucine labeling, these authors observed a ~33% reduction in hepatic protein RRs in CR compared to AL mice (Karunadharma *et al*., 2015).

#### Rapa treatment model

Hepatic proteomic analyses were initially conducted in 8‐month‐old female UM‐HET3 mice after 4 months on either a control diet or a Rapa (14 ppm) diet. We determined *k* values for 270 proteins, RPS values for 254 proteins, and WPASR values for 199 proteins in both control and Rapa (14 ppm) mice. Values of *k* were reduced for 32.6% and increased for 10.4% of the identified proteins in Rapa (14 ppm) compared to control mice. RPS values were reduced for 10.2% and increased for 31.9% of the identified proteins in Rapa (14 ppm) compared to control mice. The percentage of identified proteins that exhibited a reduction in WPASR in Rapa (14 ppm) compared to control mice was very similar to the percentage that were increased, with 24.6% exhibiting a reduction in WPASR and 27.7% exhibiting an increase (Fig. 2G–I, Table 1, and Spreadsheet S1). Mean hepatic proteome RRs were modestly but significantly reduced in Rapa (14 ppm) compared to control mice (mean ratio of Rapa (14 ppm) *k* to control *k*: 0.946; Fig. 3C and Table 1). These findings are consistent with a recent study using a similar experimental design (10 weeks of 14 ppm Rapa treatment in 3‐month‐old mice) that reported a ~13% reduction in hepatic protein RRs in Rapa‐treated compared to control mice (Karunadharma *et al*., 2015).

We found no difference in the mean WPASR in Rapa (14 ppm) compared to control mice (mean ratio of Rapa (14 ppm) WPASR to control WPASR: 1.02; Fig. 3F and Table 1). A study by another group using a different treatment regimen also reported that chronic Rapa treatment has no effect on hepatic ribosome activity in mice, an indirect metric of protein synthesis (Garelick *et al*., 2013). This is in contrast to acute Rapa treatment, which has been reported to reduce *in vivo* hepatic protein synthesis in mice (Garelick *et al*., 2013).

Taken together, these results identify the primary common global hepatic proteome alteration in all three models to be a significant reduction in protein RRs (Fig. 3A–C). A reduction in *in vivo* hepatic protein RRs (prolonged half‐lives), but not overall absolute protein synthesis rates, may therefore be an early BM of maxLS extension in mice. Importantly, this reduction in protein RRs cannot be explained by a simple reduction in cell proliferation rates. Specifically, the absolute differences in protein replacement between control and experimental groups were of a much greater magnitude (8.5%/d, 2.8%/d, and 1.1%/d) compared to the absolute differences in cell RRs (0.23%/d, 0.26%/d, and 0.02%/d) for the Snell Dwarf, CR, and Rapa models, respectively. Additionally, it is unlikely that rapidly replaced proteins and/or a specific subclass of proteins biased our observations, as proteins with far‐ranging RRs involved in a wide range of biological processes were captured (*k* value range: 1.7–72% new per day; Spreadsheet S1).

Additional common hepatic proteome alterations that were observed in all three models included a reduction in the WPASRs of proteins involved in protein processing in the endoplasmic reticulum (ER) and a preservation or increase in the WPASRs of glutathione S‐transferases (GSTs) relative to all other proteins (Figs S1 and S2). Western blot analysis revealed that chaperone levels were either unchanged or reduced in all three models (Fig. S3).

**Figure 4:**
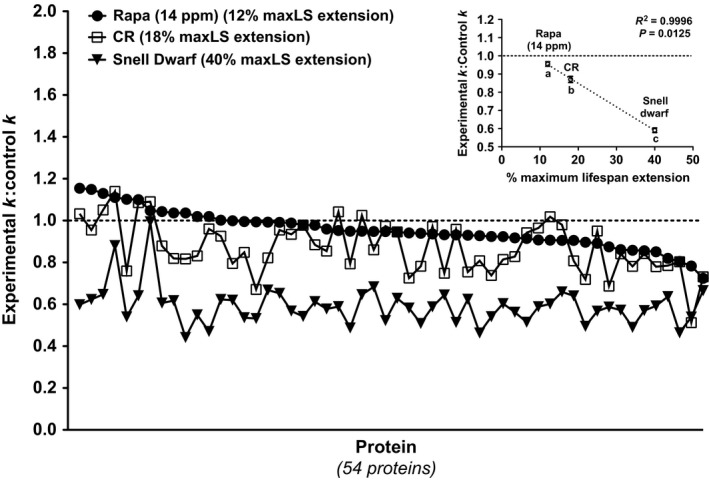
Comparison of the degree of change in the RRs (*k*) of hepatic proteins identified in all three models. Experimental *k*: control *k* ratio of each of the 54 proteins identified in all three models. Each symbol represents the experimental *k*: control *k* ratio for a given protein in a given model. For clarity, proteins are sorted in descending order based on Rapa (14 ppm) model values. The number in parentheses represents the % maxLS extension in each model. Inset: % maxLS extension vs. mean experimental *k*: control *k* ratio for the 54 proteins identified in all three models (Rapa (14 ppm) data from Rapa study 1). Values are expressed as mean ± SEM. A repeated‐measures ANOVA with Tukey's *post hoc* test was used to analyze between‐model differences (models not sharing a letter are significantly different, *P* < 0.05). *R* ^2^ and *P* values were derived from linear regression analysis. LS, lifespan. Rapa (14 ppm) data from Rapa study 1.

By comparing the 54 proteins for which we derived RR data in all three models, we found a significant correlation between the degree of maxLS extension and the degree of hepatic protein RR reduction. The degree of hepatic protein RR reduction was smallest in response to 14 ppm Rapa treatment, which is established to extend maxLS by 12%, and greatest in Snell Dwarf mice, which live 40% longer than their Het/WT counterparts, while hepatic protein RRs were reduced to an intermediate level in response to 40% CR, which extends maxLS by 18% (Fig. 4) (Blackwell *et al*., 1995; Flurkey *et al*., 2002; Harrison *et al*., 2009; Miller *et al*., 2011, 2013). These data suggest that a reduction in hepatic protein RRs may be an early BM not only qualitatively of maxLS extension but also quantitatively of the degree of maxLS extension in mice. Interestingly, the significant correlation between the degree of maxLS extension and the degree of hepatic protein RR reduction was maintained even when all proteins identified in each model were considered, not just those commonly identified in all three models (Fig. S4).

**Figure 5:**
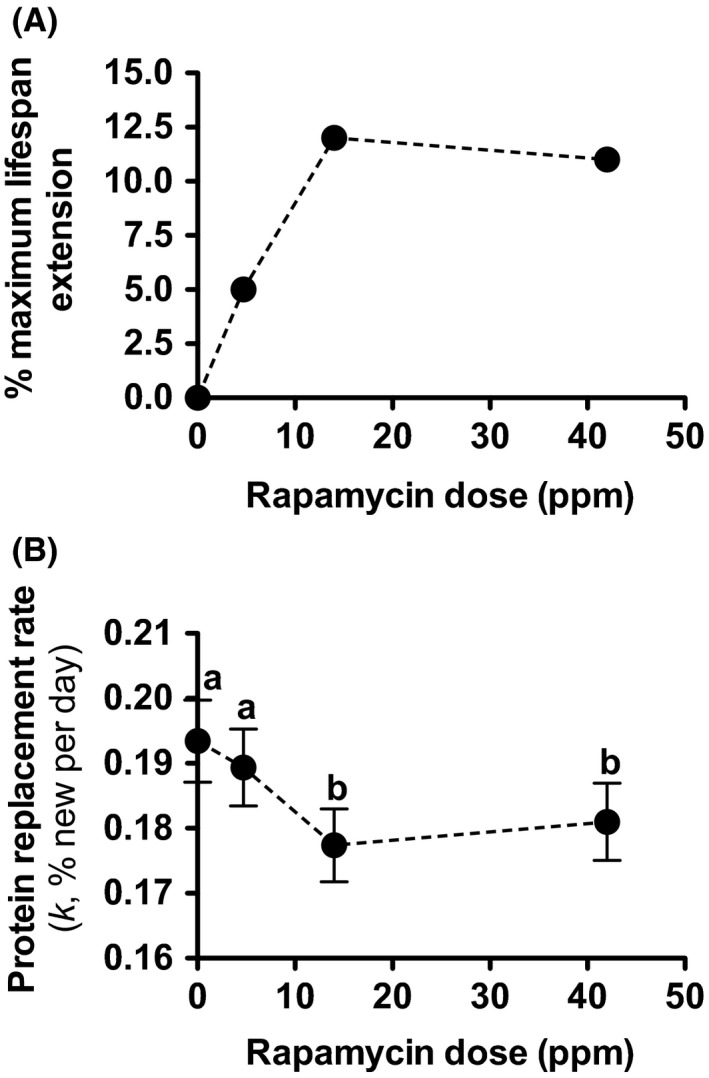
Effects of different doses of Rapa on maxLS and mean *in vivo* hepatic protein RRs (*k*). A) Rapa dose (ppm) vs. % maxLS extension (adapted from *Miller et al*.; % maxLS in response to Rapa (14 ppm) represents mean of % maxLS extensions reported in Miller *et al*., 2011, 2013) and B) Rapa dose (ppm) vs. mean hepatic protein RR (*k,* expressed as % new per day). A total of 150 proteins were identified in all four dosage groups (*n* values for each protein are provided in Spreadsheet S1). Values are expressed as mean ± SEM. A repeated‐measures ANOVA with Tukey's *post hoc* test was used to analyze between‐dose differences (doses not sharing a letter are significantly different, *P* < 0.05). Data from Rapa study 2.

To test this hypothesis further, we measured changes in hepatic protein RRs in response to three different doses of Rapa. A recent ITP study found that Rapa treatment extends maxLS in mice in a dose‐dependent manner at doses of 4.7 and 14 ppm but that there is no additional extension of maxLS at 42 ppm relative to 14 ppm (Fig. 5A; Miller *et al*., 2013). The question that we specifically addressed here was whether changes in hepatic protein RRs were predictive of the degree of maxLS extension at these different doses of Rapa and would therefore plateau at 14 ppm. We found that hepatic protein RRs were reduced in a dose‐dependent manner at the 4.7 and 14 ppm doses (reaching statistical significance only at the 14 ppm dose) but that there was no additional reduction in hepatic protein RRs at 42 ppm relative to 14 ppm (Fig. 5B and Spreadsheet S1). These dose–response data provide additional evidence that a reduction in hepatic protein RRs may be a quantitative correlate and BM of the degree of maxLS extension in mice.

A correlation is also apparent between *k* and %mean or medianLS extension across the three models, but the effect of Rapa is greater than that of CR on %mean or medianLS extension while being less than that of CR on *k,* and the correlation is not statistically significant (Fig. S5). Moreover, the plateau dose–response of Rapa on *k* at >14 ppm is not mirrored by a plateau effect on %mean or medianLS extension (Fig. S6). Accordingly, a statistically significant quantitative relationship has been demonstrated here between *k* and %maxLS extension but not between *k* and %mean or medianLS extension.

## Discussion

The major findings of this work are as follows: (i) A reduction in hepatic proteome RRs (longer half‐lives) is a common feature of all three models evaluated (Snell Dwarf, CR, and Rapa‐treated mice); (ii) a strong correlation exists between the degree to which hepatic proteome RRs are reduced and the degree of maxLS extension in these models; and (iii) *in vivo* cell proliferation rates are not consistently reduced at early time points in all these models.

The first observation could have more than one underlying cause. We do not believe that our data suggest that a reduction in hepatic proteome RRs is an initiating factor that directly promotes maxLS extension in the three models evaluated here. Rather, our hypothesis is that reduced hepatic proteome RRs reflect a reduced demand for protein renewal, and thus improved proteostasis in these models, likely due to reduced levels of misfolded and/or damaged proteins. The data presented here differentiate between several potential mechanisms of improved hepatic proteostasis in these models. In principle, a variety of cellular adaptations could reduce the levels of misfolded and/or damaged proteins, including an increase in proteolytic editing capacity, an increase in chaperone capacity, a reduction in the levels of damaging metabolites, or an increase in translational fidelity. The data presented here are not consistent with increased proteolytic editing as a major contributing factor to improved proteostasis, as reduced rather than increased proteome RRs (proteolytic rates) were consistently observed in all three models. We also found that the levels of the chaperones that we assessed were either unchanged or reduced in all three models, suggesting that an increase in chaperone capacity is an unlikely contributing factor to improved proteostasis (Fig. S3). Consistent with improved proteostasis, as well as an absence of accumulated unfolded proteins in the ER in these three models, our proteomic analyses revealed that the synthesis of proteins involved in protein processing in the ER was reduced relative to the synthesis of all other proteins in the three models (Fig. S1).

While we did not directly measure protein damage or the levels of protein‐damaging metabolites in the present studies, we did find that the only subset of related proteins that exhibited preserved or increased synthesis based on our proteomic analyses in all three models was glutathione S‐transferases (GSTs; Fig. S2). GSTs play critical protective roles in cells by inactivating molecules capable of damaging various cellular components including proteins (Hayes *et al*., 2005). It is possible that improved proteostasis in the three models evaluated in our studies is related at least in part to reduced accumulation of damaged proteins by virtue of increased or preserved GST synthesis and function.

The levels of damaged and/or misfolded proteins in cells can also be reduced by increasing translational fidelity, as mistranslated proteins are more susceptible to becoming misfolded and damaged (Ballesteros *et al*., 2001). Translational fidelity can be increased by simply slowing down the rate of translation elongation (Conn & Qian, 2013; Sherman & Qian, 2013). While we did not directly measure elongation rates here, the possibility that elongation rates may be reduced in the models evaluated here is supported by the fact that inhibition of mTOR complex 1 (mTORC1) (a feature of all three models) *in vitro* reduces the rate of elongation and increases translational fidelity (Conn & Qian, 2013).

Karunadharma *et al*. (2015) recently published an elegant study that is highly complementary to the results presented here and provides mechanistic insights into improved proteostasis in two of the three models evaluated here. Using heavy‐leucine labeling, this group found that 10 weeks of 40% CR in 25‐month‐old mice and 10 weeks of 14 ppm Rapa treatment in 3‐month‐old mice reduced hepatic protein RRs by 30% and 13%, respectively. Furthermore, they provided evidence of reduced hepatic protein damage in both CR and Rapa‐treated mice and indirect evidence of reduced translation elongation rates in Rapa‐treated (but not CR) mice. These data provide evidence that the reduction in hepatic protein RRs in CR and Rapa‐treated mice is due to a reduced demand for protein renewal attributable to a reduction in protein damage, consistent with the model that we propose here. Reduced protein damage has been previously reported in Snell Dwarf mice relative to control counterparts (Flurkey *et al*., 2001); however, it remains to be determined whether elongation rates are reduced and translational fidelity is increased in these mice.

The observation that protein RRs (i.e., proteolytic clearance rates) are reduced in the three models evaluated is perhaps counterintuitive in light of reports of reduced mTORC1 signaling and enhanced autophagy in these models (Hsieh & Papaconstantinou, 2004; Wang & Miller, 2012; Fok *et al*., 2013). Autophagy is a lysosomal pathway that degrades various cellular components, including proteins and organelles, and is suppressed by mTORC1 signaling and upregulated in response to certain forms of stress, such as nutrient deprivation (Klionsky & Emr, 2000). Upregulation of autophagy would be expected to increase protein RRs, but we reproducibly measured a general reduction in hepatic proteome RRs in all three models. One possible explanation for this observation is that flux through the autophagic pathway is in fact not enhanced in these models, as many researchers rely on surrogate as opposed to direct measurements of autophagic flux.

It is important to note the limitations of the present study. First, our main conclusion that a reduction in hepatic protein RRs may serve as a BM of maxLS extension is based on the assessment of only three models. We believe that continued validation of this putative BM is required in other models of maxLS extension. In particular, this putative BM should be tested in models that are not as intimately linked to reduced nutrient sensing and altered mTORC1 signaling as the three models assessed here. Given that maxLS can likely be extended via multiple mechanisms, we do not expect reduced hepatic protein RRs, or any other single BM, to manifest in response to all interventions that extend maxLS. A reduction in hepatic protein RRs might serve as one BM among a panel, however. Second, there was variability in the duration of heavy water labeling period in our studies. However, in all three models, proteins with a wide range of RRs were identified [2.1–72.4% in Snell Dwarf, 8.5–54.4% in CR, and 1.7–63.9% in Rapa (14 ppm)] across multiple gene ontologies, so a bias toward fast or slow turnover proteins or a functional class of proteins is not likely. Third, our main conclusion is based on the kinetic assessment of a modest number or proteins, and the data presented may be biased toward more abundant proteins in liver. Assessing the RRs of only a subset of hepatic proteins of relatively high abundance may, however, capture the key information regarding proteostasis in these settings. Lastly, there was variability in the age of assessment and duration of intervention. Importantly, all mice were assessed at or before reaching their half‐maxLS. The duration of intervention was 21–27%, 40%, and 11% of maxLS in the Snell Dwarf, CR, and Rapa (14 ppm) models, respectively. Despite this variability, we observed a significant correlation between the degree of hepatic protein RR reduction and maxLS extension at relatively early time points within the LS of each model. Moreover, demonstration of a dose–response relationship is generally considered a particularly strong class of supportive evidence in pharmacologic studies. Thus, the value of reduced hepatic protein RRs as a BM is bolstered by the Rapa dose–response study in which we observed that *within* a given model, a reduction in hepatic protein RRs is a strong and sensitive predictor of the degree of maxLS extension.

Overall, the data presented here demonstrate that a reduction in hepatic protein RRs meets at least 3 of the 4 criteria listed above for an early rate‐based BM of maxLS extension, as this BM (i) manifests relatively soon after the initiation of each intervention (after 4–14 months of intervention, corresponding to 11–40% of total LS); (ii) is predictive of maxLS extension at early time point measurements; (iii) is not strain‐ or intervention‐specific (three interventions were tested each on a unique genetic background) (although both sexes were not tested); and (iv) reveals the activity (i.e., flux) of a targeted biological pathway (proteome replacement). As part of future efforts to continue to validate reduced hepatic protein RRs as a BM of maxLS extension, it will be important to determine whether a reduction in hepatic protein RRs predicts maxLS extension at earlier time points and in both sexes of the models presented here. A good test of the validity of this BM would be to determine whether it recapitulates the well‐established sex‐specific difference in the degree of maxLS extension in response to Rapa treatment.

Measuring hepatic protein RRs in response to interventions proven to have no effect on maxLS in mice, or only on meanLS, will also be necessary to determine the extent to which this putative BM is specific for maxLS extension and thus, potentially, a slowing of the aging process.

It is possible that the observed effect on proteome RRs in the liver in these three models manifests in other tissues, including blood plasma, which can be accessed relatively noninvasively. Translation of this BM approach in these models may therefore be possible by measuring plasma protein RRs, a technique already established in humans (Price *et al*., 2012a).

In summary, the quest to extend human healthspan requires the development of a panel of early and sensitive BMs of maxLS extension for prioritization of candidate interventions into large‐scale healthspan and LS studies in mice. While further validation will be essential, the data presented here provide evidence that a reduction in global hepatic proteome RRs may serve as one such BM.

## Experimental procedures

### Mice, animal husbandry, diets, feeding regimens, and duration of heavy water (^2^H_2_O) labeling

Details regarding the published %maxLS extension, groups, strain, sex, age at initiation of intervention, duration of intervention, and duration of heavy water labeling for each model for each type of study can be found in Table 2. All other details regarding husbandry, diets, feeding regimens, and duration of heavy water labeling are presented in Supporting Information. All experiments were performed under the approval of the Institutional Animal Care and Use Committees of the University of California at Berkeley.

**Table 2:** Summary of studies performed here and of previously reported studies

Model	Type of intervention	Study	Previously reported % maximum lifespan extension^a^	Groups	Strain (Sex)	Age at initiation of intervention	Duration of intervention	^2^H_2_O label duration (days)	kDa range of proteins analyzed
Snell Dwarf	Monogenetic^b^	CP	40% (Flurkey *et al*., 2002)	Het/WT or Snell Dwarf	DW/J (F)	Birth	6–7 months^c^	19	NA
		P	40% (Flurkey *et al*., 2002)	Het/WT or Snell Dwarf	DW/J (F)	Birth	5.5–7 months^c^	1, 2, 4	10–80
CR	Dietary	CP	ND	AL or CR^d^	C57BL/6 (F)	4 months	6 weeks	20	NA
		P^e^	18% (Blackwell *et al*., 1995)	AL or CR^f^	C57BL/6 (M)	4 months	14 months	1, 2, 4, 8, 15, 32	3.5–160
Rapa Treatment	Pharmacological	CP and P^g^	12% (14 ppm) (Miller *et al*., 2011, 2013)^h^	Control or Rapa	UM‐HET3 (F)	4 months	4 months	2^i^, 6, 18, 24	3.5–160
		P^j^	5% (4.7 ppm) (Miller *et al*., 2013) 12% (14 ppm) (Miller *et al*., 2011, 2013)^h^ 11% (42 ppm) (Miller *et al*., 2013)	Control or Rapa	UM‐HET3 (F)	4 months	4 months	2	20–40

a Relative to control counterparts.

b Homozygous for loss‐of‐function recessive mutation in Pit1 gene.

c Duration of intervention taken to be the age of the animals at the time of euthanasia.

d 25% calorie‐restricted relative to AL.

e Adapted from (Price *et al*., 2012b).

f 40% calorie‐restricted relative to AL.

g Rapa proteomics study 1.

h Mean of % maximum lifespan extensions reported in Miller *et al*. (2011, 2013).

i 2‐day‐labeled mice used for Rapa proteomics study 1.

j Rapa proteomics study 2.

CP, cell proliferation; P, proteomics.

### Heavy water labeling protocols

To measure rates of *in vivo* DNA synthesis and protein replacement, mice were labeled with an intraperitoneal injection of 100% ^2^H_2_O at the times specified in Table 2 prior to the end of each study. Mice were then provided free access to 8% heavy water as drinking water for the remainder of the studies, as previously described (Bruss *et al*., 2011).

### LC‐MS/MS analysis

Details of LC‐MS/MS analysis methods are presented in Supporting Information. Details regarding specific peptides and proteins identified and unique peptide coverage are presented in Spreadsheets S1 and S2.

### Calculation of fractional replacement (*f*), replacement rate constant (*k*), relative pool size (RPS), and within‐proteome absolute synthesis rates (WPASR) for individual proteins

Details of *f* calculations were described previously (Price *et al*., 2012a), and calculation of *k*, RPS, and WPASR values for individual proteins is presented in Supporting Information. Individual protein‐level *k*, RPS, and WPASR data and individual peptide‐level *f* and RPS data are presented in Spreadsheet S1.

### Statistical analyses

Data were analyzed using GraphPad Prism software (version 5.0a) (La Jolla, CA, USA) and Real Statistics Resource Pack (http://www.real-statistics.com/free-download/real-statistics-resource-pack/) in Excel (2011).

All other experimental procedures are presented in Supporting Information.

## Author contributions

ACST, MDB, JCP, CFK, DEH, and MKH conceived and designed experiments. ACST, MDB, JCP, CFK, MD, and LSR performed experiments. ACST, MDB, JCP, CFK, WEH, and MC analyzed data. JCP, WEH, MC, CMA, DEH, and MKH contributed reagents/materials/analysis tools. ACST, MDB, JCP, CFK, DEH, and MKH contributed to writing the manuscript.

## Conflict of interest

None declared.

## Funding

This work was supported by NIH grant AG034297‐01 and College of Natural Resources, University of California at Berkeley, support to MKH; funds from KineMed, Inc.; and NIH grants AG038560, AG022308, and AG032333 to DEH plus The Jackson Laboratory's CA034196 (for work at TJL).

## Supporting information


**Spreadsheet S1.** GO terms, protein‐level data and peptide‐level data for each model.

**Spreadsheet S2.** Spectrum Mill protein identification details.

**Fig. S1.** Hepatic synthesis of proteins involved in protein processing in the ER (PPER).
**Fig. S2.** Hepatic synthesis of GST proteins.
**Fig. S3.** Chaperone levels in the liver.
**Fig. S4.** Correlation of % maxLS extension and change in hepatic protein replacement rates (*k*) across models.
**Fig. S5.** Correlation of % meanLS or % medianLS extension and change in hepatic protein replacement rates (*k*) across models.
**Fig. S6.** Effects of different doses of rapamycin on % medianLS and *in vivo* hepatic protein replacement rates (*k*).
**Data S1.** Additional experimental procedures.
