# Supplementary material for: Reduced in vivo hepatic proteome replacement rates but not cell proliferation rates predict maximum lifespan extension in mice
Source: Aging Cell. 2015 Nov 6;15(1):118–27. doi: 10.1111/acel.12414 (PMC4717272; doi:10.1111/acel.12414)
Supplement: Supplementary file 3 — Fig. S1. Hepatic synthesis of proteins involved in protein processing in the ER (PPER). Fig. S2. Hepatic synthesis of GST proteins. Fig. S3. Chaperone levels in the liver. Fig. S4. Correlation of % maxLS extension and change in hepatic protein replacement rates (k) across models. Fig. S5. Correlation of % meanLS or % medianLS extension and change in hepatic protein replacement rates (k) across models. Fig. S6. Effects of different doses of rapamycin on % medianLS and in vivo hepatic protein replacement rates (k). Data S1. Additional experimental procedures. [file ACEL-15-118-s003.docx]

**Supporting Information:**

**Reduced *in vivo* hepatic proteome replacement rates but not cell proliferation rates predict maximum lifespan extension in mice**

Airlia C. S. Thompson, Matthew D. Bruss, John C. Price, Cyrus F. Khambatta, William E. Holmes, Marc Colangelo, Marcy Dalidd, Lindsay S. Roberts, Clinton M. Astle, David E. Harrison and Marc K. Hellerstein

**Spreadsheet Descriptions**

**SI Spreadsheet 1. GO terms, protein-level data and peptide-level data for each model.** For each model, gene ontology biological process terms, *k* values, relative pool sizes (RPS) and within proteome absolute synthesis rates (WPASR) are provided for each protein. Corresponding peptide-level fractional synthetic rate (*f*) and RPS values are also provided for each model. The following is a description of the information contained within each tab:

Tab 1: GO BP terms for Snell Dwarf model

Tab 2: Protein-level *k*, RPS and WPASR data for Snell Dwarf model

Tab 3: Peptide-level *f* data for Snell Dwarf model

Tab 4: Peptide-level RPS data for Snell Dwarf model

Tab 5: GO BP terms for CR model

Tab 6: Protein-level *k*, RPS and WPASR data for CR model

Tab 7: Peptide-level *f* data for CR model

Tab 8: Peptide-level RPS data for CR model

Tab 9: GO BP terms for Rapamycin model (Rapamycin study 1)

Tab 10: Protein-level *k*, RPS and WPASR data for Rapamycin model (Rapamycin study 1)

Tab 11: Peptide-level *f* data for Rapamycin model (Rapamycin study 1)

Tab 12: Peptide-level RPS data for Rapamycin model (Rapamycin study 1)

Tab 13: Protein-level *k* data for Rapamycin model (Rapamycin study 2)

Tab 14: Peptide-level *f* data for Rapamycin model (Rapamycin study 2)

**SI Spreadsheet 2. Spectrum Mill protein identification details.** For each protein the accession number, name, number of spectra, number of unique peptides and percent coverage are provided. Note: This spreadsheet contains some proteins that did not meet certain filtering criteria and, therefore, were not included in final datasets.

**Figures**

**SI Figure 1. Hepatic synthesis of proteins involved in protein processing in the ER (PPER).** Comparisons between the within proteome absolute synthesis rate (WPASR) of proteins involved in PPER in A) Snell Het/WT vs. Dwarf (n = 3-6 per group, total of 4 PPER proteins), B) AL vs. CR (n = 4-9 per group, total of 4 PPER proteins) and C) control vs. Rapa (14 ppm) mice (n = 3 per group, total of 11 PPER proteins). Values are expressed as the mean ± SEM. Student’s paired two-tailed *t*-tests were used for all between-group analyses (^ p = 0.077, ** p < 0.006, *** p < 0.0001). Experimental WPASR to control WPASR ratio for proteins involved in PPER compared to all other proteins identified in the D) Snell Dwarf (total of 4 PPER proteins and 66 other proteins), E) CR (total of 4 PPER proteins and 80 other proteins) and F) Rapa (14 ppm) model (total of 11 PPER proteins and 188 other proteins). Values are expressed as the mean ± SEM. Student’s unpaired two-tailed *t*-tests with Welch’s correction were used for all between-group analyses (* p < 0.028, *** p < 0.0001). An in-house Python script was developed to determine which Kyoto Encyclopedia of Genes and Genomes (KEGG) pathway(s) identified proteins mapped to. PPER proteins identified include: 78 kDa glucose-regulated protein (UniProt accession #P20029, identified in Rapa (14 ppm), CR and Snell Dwarf models), 94 kDa glucose-regulated protein (UniProt accession #P08113, identified in Rapa (14 ppm) model), Glucosidase 2 subunit beta (UniProt accession #O08795, identified in Rapa (14 ppm) model), Heat shock cognate 71 kDa protein (UniProt accession #P63017, identified in Rapa (14 ppm), CR and Snell Dwarf models), Heat shock protein HSP 90-alpha (UniProt accession #P07901, identified in Rapa (14 ppm) model), Heat shock protein HSP 90-beta (UniProt accession #P11499, identified in Rapa (14 ppm) model), Hypoxia up-regulated protein 1 (UniProt accession # Q9JKR6, identified in Rapa (14 ppm) model), Neutral alpha-glucosidase AB (UniProt accession #Q8BHN3, identified in Rapa (14 ppm) model), Protein disulfide-isomerase (UniProt accession # P09103, identified in Rapa (14 ppm), CR and Snell Dwarf models), Protein disulfide-isomerase A3 (UniProt accession #P27773, identified in Rapa (14 ppm), CR and Snell Dwarf models) and Transitional endoplasmic reticulum ATPase (UniProt accession #Q01853, identified in Rapa (14 ppm) model)

**SI Figure 2. Hepatic synthesis of GST proteins.** Comparisons between the within proteome absolute synthesis rate (WPASR) of GSTs in A) Snell Het/WT vs. Dwarf (n = ­2-4 per group, total of 5 GSTs), B) AL vs. CR (n = ­3-10 per group, total of 4 GSTs) and C) control vs. Rapa (14 ppm) mice (n = 2-3 per group, total of 5 GSTs). Values are expressed as the mean ± SEM. Student’s paired two-tailed *t*-tests were used for all between-group analyses (* p < 0.011, ** p < 0.006). Experimental WPASR to control WPASR ratio for GSTs compared to all other proteins identified in the D) Snell Dwarf (total of 5 GST proteins and 65 other proteins), E) CR (total of 4 GST proteins and 80 other proteins) and F) Rapa (14 ppm) model (total of 5 GST proteins and 194 other proteins). Values are expressed as the mean ± SEM. Student’s unpaired two-tailed *t*-tests with Welch’s correction were used for all between-group analyses (^ p = 0.167, * p < 0.013, *** p < 0.0001). An in-house Python script was developed to determine which Kyoto Encyclopedia of Genes and Genomes (KEGG) pathway(s) identified proteins mapped to. GST proteins identified include: Glutathione S-transferase A1 (UniProt accession #P13745, identified in CR and Snell Dwarf models), Glutathione S-transferase A3 (UniProt accession #P30115, identified in Rapa (14 ppm), CR and Snell Dwarf models), Glutathione S-transferase A4 (UniProt accession #P24472, identified in Rapa (14 ppm) model), Glutathione S-transferase Mu 1 (UniProt accession #P10649, identified in Rapa (14 ppm), CR and Snell Dwarf models), Glutathione S-transferase Mu 2 (UniProt accession #P15626, identified in Rapa (14 ppm) and Snell Dwarf models), Glutathione S-transferase Mu 7 (UniProt accession #Q80W21, identified in Snell Dwarf model) and Glutathione S-transferase P 2 (UniProt accession #P19157, identified in Rapa (14 ppm) and CR models).

**
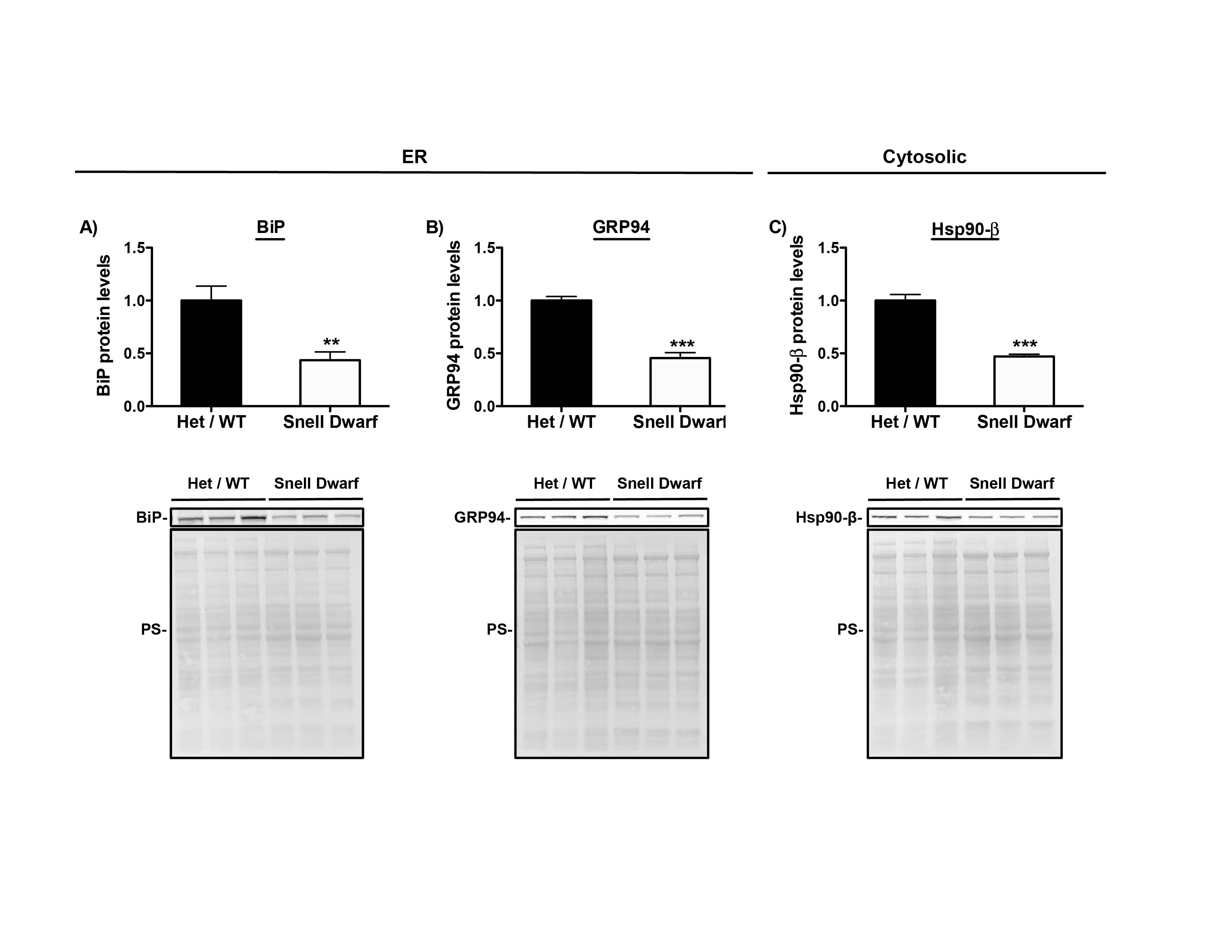
**

**
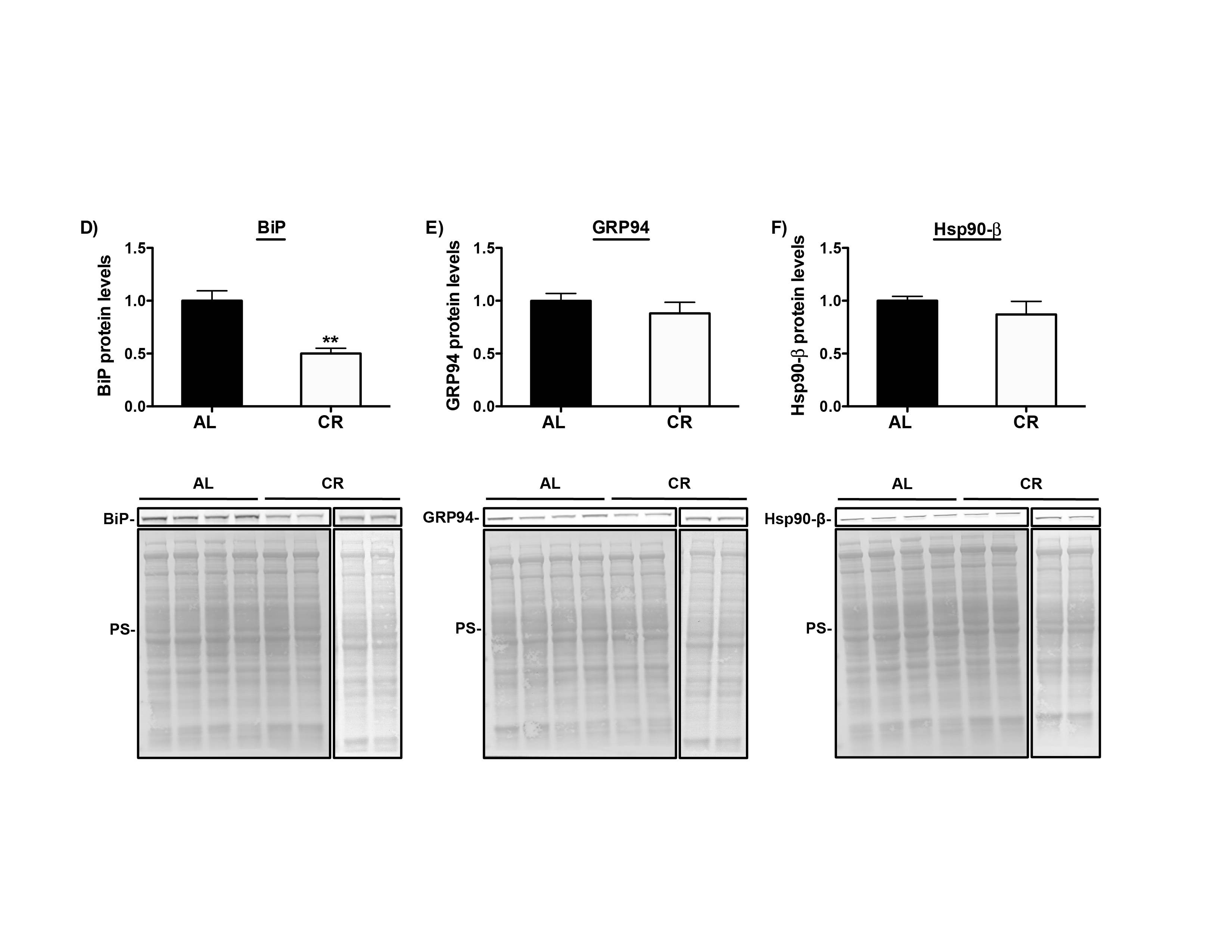
**

**
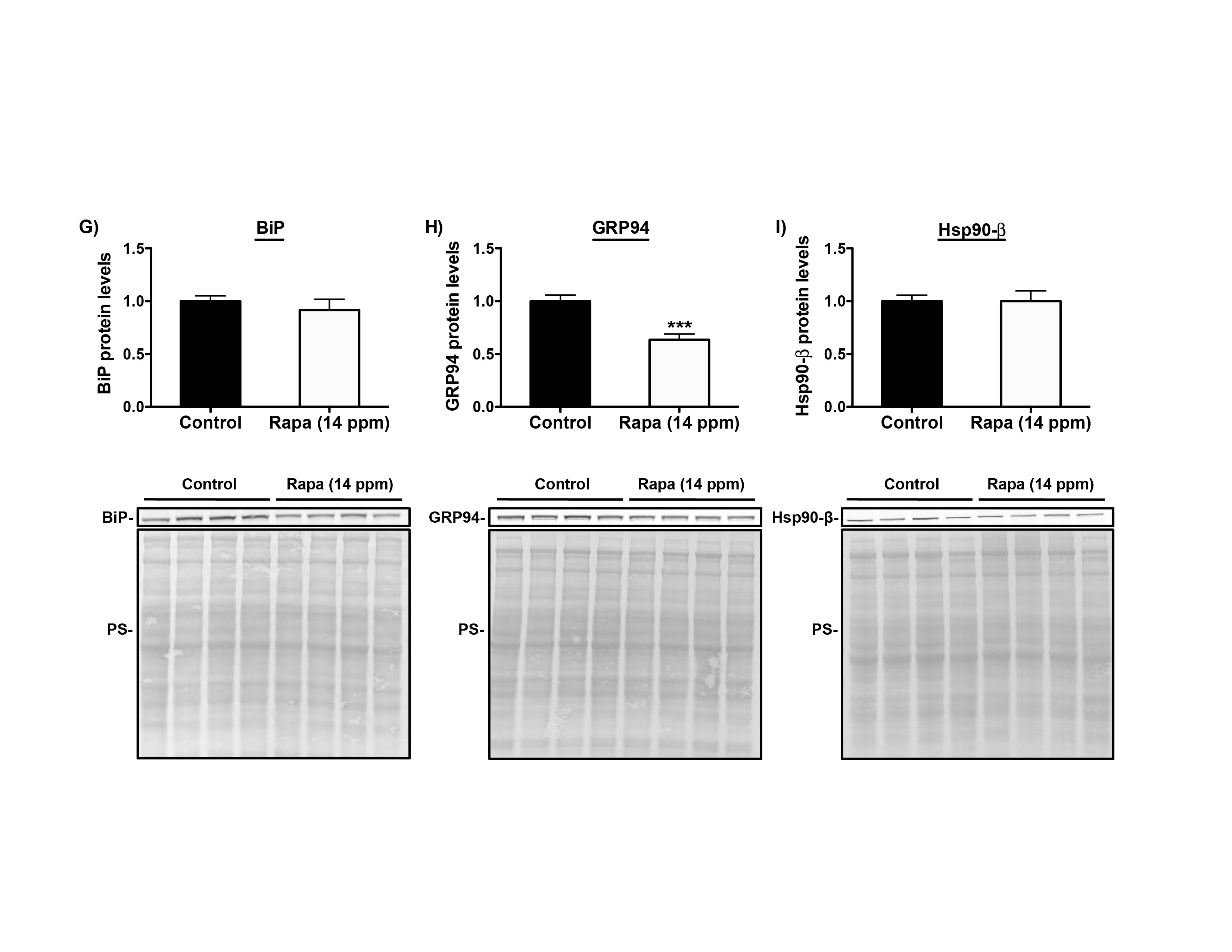
**

**SI Figure 3. Chaperone levels in the liver.** Western blot images, Ponceau S (PS) stain images and corresponding normalized densitometry for levels of hepatic BiP, GRP94 and Hsp90-β in A-C) Snell Het/WT vs. Dwarf (n = 5-6 per group), D-F) AL vs. CR (n = 4-8 per group) and G-I) control vs. Rapa (14 ppm) mice (n = 8-12 per group). Total protein within a given lane, as determined by PS staining, was used as the loading control. Values are normalized to control counterpart group within each model. Values are expressed as the mean ± SEM. Student’s unpaired two-tailed *t*-tests were used for all between-group analyses (** p < 0.006, *** p < 0.0001). Frozen livers were homogenized in 350-400ul lysis buffer (10mM Tris-base, 150mM NaCl, 1% NP-40, 0.1% SDS, 0.5% sodium deoxycholate, 1mM DTT, 1mM PMSF, 7.5ug/mL leupeptin, 1.0ug/mL pepstatin, 2.0ug/mL aprotinin and 1 Phosphatase Inhibitor Cocktail tablet (Roche Applied Science, Indianapolis, IN) per 10mL buffer, pH ~7.5) using a stainless steel bead and a TissueLyserII (Retsch, Newtown, PA) set at 30hz for 1 min. Tissue homogenates were tip-sonicated on ice for 3 x 15sec pulses at 10% amplitude with 10sec pauses in between pulses. Protein concentrations were determined by BCA assay (Pierce, Rockford, IL) and proteins were separated by SDS-PAGE (Invitrogen, Grand Island, NY). Prior to blocking, all membranes were incubated in ~15mL 0.1% Ponceau S (PS) (w/v) and 5.0% acetic Acid (w/v) for 15-20min. Membranes were then partially destained in deionized water to bring out PS stained bands before imaging. Membranes were then completely destained via incubation in 0.1M NaOH for 30sec followed by 2 minutes of rinsing under running deionized water. Following blocking, membranes were immunoblotted with primary antibodies against glucose-regulated protein 78 (GRP78, also known as BiP), GRP94 and heat shock protein 90-β (Hsp90-β) (Cell Signaling, Danvers, MA) followed by incubation with IRDye®700DX-conjugated secondary antibodies (Rockland Immunochemicals Inc., Gilbertsville, PA). BiP, GRP94 and Hsp90-β protein bands were imaged and densitometry measurements were made using the Odyssey® Infrared Imaging System (LI-COR, Lincoln, NE). The total amount of protein loaded per lane was used as a loading control. Total protein per lane was quantified by taking densitometry measurements of PS staining for each lane using Image J (National Institutes of Health, Bethesda, Maryland).

**SI Figure 4. Correlation of % maxLS extension and change in hepatic protein replacement** **rates (*k*) across models.** The % maxLS extension vs. the mean experimental *k* : control *k* ratio is plotted for each model when all proteins identified in each model are considered (open circles with solid trend line) as well as when only the 54 proteins commonly identified in all three models are considered (closed triangles with dashed trend line) (Rapa (14ppm) data from rapamycin study 1). Values are expressed as the mean ± SEM. R^2^ and p values were derived from linear regression analysis.

**SI Figure 5. Correlation of % meanLS or % medianLS extension and change in hepatic protein replacement** **rates (*k*) across models.** % meanLS or % medianLS extension vs. mean experimental *k* : control *k* ratio for the 54 proteins identified in all three models (Rapa (14ppm) data from rapamycin study 1). Values are expressed as the mean ± SEM. R^2^ and p values were derived from linear regression analysis. LS = lifespan. % LS extension values for the Snell Dwarf (Flurkey et al. 2002) (48%) and CR (Blackwell et al. 1995) (15.4%) models represent reported % meanLS extension values. % LS extension value for the rapamycin model represents the average reported % medianLS extension value derived from two separate studies (Miller et al. 2011; Miller et al. 2013) (16%).

**SI Figure 6. Effects of different doses of rapamycin on % medianLS and *in vivo* hepatic protein replacement** **rates (*k*).** A) Rapamycin dose (ppm) vs. % medianLS extension (adapted from *Miller et al.*) (% LS in response to Rapa (14ppm) represents mean of % median LS extensions reported in Miller et al. 2011 and Miller et al. 2013). B) Rapamycin dose (ppm) vs. mean protein replacement rate (*k,* expressed as % new per day). A total of 150 proteins were identified in all four dosage groups (n values for each protein are provided in **SI Spreadsheet 1**). Values are expressed as the mean ± SEM. A repeated measures ANOVA with Tukey *post hoc* test was used to analyze between-dose differences (doses not sharing a letter are significantly different, p < 0.05). Data from rapamycin proteomics study 2.

**Additional experimental procedures**

**Mice, animal husbandry, diets, feeding regimens and duration of heavy water (^2^H_2_O) labeling**

All mice in all studies were maintained under temperature- and light-controlled conditions (12h:12h light-dark cycle, lights on at 0700h and off at 1900h).

*Snell Dwarf model:* For Snell Dwarf studies, female heterozygous (Het), wild type (WT) or homozygous (Dwarf) DW/J Snell mice were purchased from the NIA Mutant Mouse Aging Colony (Taconic line number 3623). Het and WT Snell mice are phenotypically indistinguishable and, therefore, were combined into one group, Het/WT. For all studies, two Het/WT mice were caged with two Dwarf mice and mice were provided with NIH-41 diet in pellet and powdered form (in a dish placed on the top of the bedding in each cage). The body weight of each mouse was measured one to three times per week. For the *in vivo* cell proliferation study, ~5- to 6-month old female Het/WT and Snell Dwarf mice were used. Mice in this study were labeled with heavy water for the last 19 days of the study. For the *in vivo* hepatic proteomics study, ~4.5- to 6-month old female Het/WT and Snell Dwarf mice were used. Mice in this study were labeled with heavy water for the last 1, 2, or 4 days of the study.

*CR model:* For the *in vivo* cell proliferation study, 4-month-old female C57BL/6 mice (Charles River, Wilmington, MA) were used. All mice were housed individually. Mice were randomly assigned to one of the following two groups: *ad libitum*-fed (AL) or CR. Mice in the AL group were provided unrestricted access to the NIH41 diet (Diet# 58YP, TestDiet, St. Louis, MO). Due to excessive powdering of the NIH41 diet, which prohibited the accurate measurement of food intake in the AL group, mice in the CR group were provided with enough NIH41-fortified diet (Diet# 5TPD, TestDiet, St. Louis, MO) to achieve a 25% reduction in body weight relative to the AL group mean by week 3 of the study. Mice in the CR group were then provided with enough NIH41-fortified diet to maintain a body weight that was 75% of the AL group mean for the remaining 3 weeks of the study. Therefore, the CR mice in this study were effectively on a 25% CR diet. CR mice were provided with food daily at 1200hr. All mice in this study were kept on their diets for a total of 6 weeks. The body weight of each mouse was measured three times per week and all mice were labeled with heavy water for the last 20 days of the study. The *in vivo* hepatic proteomics data presented here for the CR model were adapted from a previous publication from our group (Price et al. 2012), however, the data reported here include an additional heavy water labeling time point, new relative protein pool size analyses and a detailed comparison of hepatic proteome alterations in Snell Dwarf, CR and rapamycin-treated mice. For these *in vivo* hepatic proteomics studies, 18-month-old male AL and CR C57BL/6 mice were purchased from Charles River (Wilmington, MA), where the NIA Caloric Restricted Mouse Colony is maintained. All mice were housed individually. Mice in the CR group had been on a 40% CR diet since 4 months of age. Mice in this study were labeled with heavy water for the last 1, 2, 4, 8, 15 or 32 days of the study. 2 day labeled AL mice were provided unrestricted access to the NIH41 diet and 2 day labeled CR mice were provided with 3.0g of the NIH41-fortified diet (both diets from Charles River, Wilmington, MA) daily between 0600hr and 0930hr. For all other labeling groups, AL mice were provided unrestricted access to the NIH31 diet and CR mice were provided with 3.0g of the NIH31/NIA fortified diet (both diets from Charles River, Wilmington, MA) daily at 1700hr. The body weight of each mouse was measured at least once per week.

*Rapamycin treatment model:* The same genetically heterogeneous mouse strain (UM-HET3) and rapamycin-containing diets used in the National Institute on Aging’s Interventions Testing Program’s lifespan studies were used in the studies presented here (Harrison et al. 2009; Miller et al. 2011; Miller et al. 2013). Specifically, 4-month-old female UM-HET3 mice were used for all studies. Mice were housed 3-5 per cage and the body weight of each mouse was measured once per week. A total of two independent rapamycin treatment studies were conducted. In the first study, mice were randomly assigned to one of the following two groups: control diet or diet containing 14ppm rapamycin (Rapa (14 ppm)). All mice in this study remained on their diets for a total of 4 months and were labeled with heavy water for the last 2, 6, 18 or 24 days of the study. *In vivo cell* proliferation rates were measured in mice from this first study. Mice in this study labeled for 2 days were also used for the initial rapamycin *in vivo* hepatic proteomics study. In the second study, mice were randomly assigned to one of the following four groups: control diet or diet containing 4.7 ppm (Rapa (4.7 ppm)), 14 ppm (Rapa (14 ppm)) or 42 ppm (Rapa (42 ppm)) rapamycin. All mice in this study remained on their diets for a total of 4 months and were labeled with heavy water for the last 2 days of the study. Mice in this second study were used for the follow-up rapamycin dose-response *in vivo* hepatic proteomics study.

**Blood, plasma and tissue collection and cell isolation**

Upon completion of each study, mice were anesthetized under 3% isoflurane and blood was collected via cardiac puncture, followed by cervical dislocation, tissue collection and in some cases cell isolation. Following centrifugation of blood, plasma was collected and stored at -20°C. Upon dissection, the liver was cut into several small pieces (~20-100mg), which were flash frozen in liquid nitrogen. For DNA synthesis measurements, liver samples were homogenized and total DNA from all liver cells was isolated. Epidermal, mammary and bone marrow cells were isolated and DNA was isolated from these cell types as previously described (Bruss et al. 2011).

**Measurement of DNA synthesis (cell proliferation)**

The fractional replacement (*f*, fraction newly divided cells) of epidermal, liver and mammary cells was determined by measuring the incorporation of ^2^H into purine deoxyribose (dR) of DNA, as previously described (Bruss et al. 2011). The fractional replacement rate (*k*, fraction newly divided cells per day) was calculated as:

where t is the number of days a given mouse was labeled with heavy water.

**Measurement of ^2^H_2_O enrichment in body water**

Enrichment of ^2^H_2_O in body water (blood) was measured via chemical conversion to tetrabromoethane as previously described (Price, et al. 2012). Body water ^2^H_2_O enrichment values (p) were used to calculate the fractional synthetic rate (*f*) of peptides as detailed in a subsequent section.

**Preparation of liver samples for LC-MS/MS proteomic analysis**

Frozen livers from AL and CR mice labeled with heavy water for 1, 4, 8, 15, or 32 days were prepared and treated as previously described (Price et al. 2012). Frozen livers from AL and CR mice labeled with heavy water for 2 days as well as livers from mice in all Snell Dwarf and rapamycin studies were homogenized in ~500ul lysis buffer (10mM Tris-base, 150mM NaCl, 1% NP-40, 0.1% SDS, 0.5% sodium deoxycholate, 1mM dithiothreitol (DTT), 1mM phenylmethylsulfonyl fluoride (PMSF), 7.5ug/mL leupeptin, 1.0ug/mL pepstatin, 2.0ug/mL aprotinin and 1 Phosphatase Inhibitor Cocktail (Roche Applied Science, Indianapolis, IN) per 10mL buffer, pH ~7.5) using a stainless steel bead and a TissueLyserII (Retsch, Newtown, PA) set at 30hz for 1min. Tissue homogenates were sonicated in a sonication water bath for 1min and then centrifuged at 10,000 rcf at 4°C for 10min followed by supernatant collection. Protein concentrations were determined by bicinchoninic acid (BCA) assay (Pierce, Rockford, IL). 100-200ug aliquots of protein from these homogenates were uniformly reduced via incubation in 4.8mM tris(2-carboxyethyl)phosphine (TCEP) and SDS-PAGE sample loading buffer for 10min at 70°C. The reduced samples were then alkylated via incubation in 14.3mM iodoacetamide for 1hr in the dark at room temperature. Tryptic peptides from all protein homogenates were prepared as previously described (Price, et al. 2012) (see **Table 2** for kDa range of proteins analyzed for each study).

In order to quantify the relative concentrations of proteins in the livers of each mouse, liver homogenates were spiked with an exogenous heavy-labeled standard (**S**table **I**sotope **LA**beling in **M**ammals, SILAM). The exogenous heavy-labeled standard used was a homogenate derived from the liver of a C57BL/6 mouse metabolically labeled with ^15^N (MouseExpress Liver, Cambridge Isotope Laboratories, Inc., Tewksbury, MA). Briefly, equal ug amounts of protein derived from the liver homogenates of mice in our studies and protein derived from the SILAM liver were combined prior to protein treatment, fractionation and LC-MS/MS analysis.

**LC-MS/MS analysis**

Trypsin-digested peptides were analyzed on either an Agilent 6520 or 6550 Q-TOF (quadrupole time-of-flight) mass spectrometer with 1260 Chip Cube nano ESI source (Agilent Technologies, Santa Clara, CA). Peptides were separated chromatographically using a Polaris HR chip (Agilent #G4240-62030) consisting of a 360nL enrichment column and a 0.075 x 150 mm analytical column, each packed with Polaris C18-A stationary phase with 3 µm particle size. Mobile phases were (A) 5% v/v acetonitrile and 0.1% formic acid in deionized water and (B) 95% acetonitrile and 0.1% formic acid in deionized water. Peptides were eluted at a flow rate of 350 nL/min during an 18 min nano LC gradient (2% B at 0 min, 5% B at 0.5 min, 30% B at 10 min, 50% B at 13 min, 90% B at 13.1-18 min, 2% B at 18.1 min; Stop time: 32 min). Each sample was analyzed twice, once for protein/peptide identification in data-dependent MS/MS mode and once for peptide isotope analysis and SILAM quantitation analysis in MS-only mode.

Acquisition parameters were: MS/MS acquisition rate = 6 Hz MS and 4 Hz MS/MS with up to 12 precursors per cycle, MS acquisition rate = 0.9 Hz (6520 QTOF) or 0.6 Hz (6550 QTOF), ionization mode = positive electrospray; capillary voltage = 1980 V; drying gas flow = 9 L/min (6520 QTOF) or 11 L/min (6550 QTOF); drying gas temperature = 290 °C; fragmentor = 360 V; skimmer = 45 V; maximum precursor per cycle = 12; scan range = 100-1700 m/z (MS), 50-1700 m/z (MS/MS); isolation width (MS/MS) = medium (~4 m/z); collision energy (V) = -4.8+3.6 (precursor m/z/100); active exclusion enabled (exclude after 1 spectrum, release after 0.12 min); charge state preference = 2, 3, >3 only, sorted by abundance; TIC target = 25,000; reference mass = 922.009798 m/z (6520 QTOF) or 1221.990637 m/z (6550 QTOF). Acquired MS/MS spectra were extracted and searched using Spectrum Mill Proteomics Workbench software (version B.04.00 released Feb 2012, Agilent Technologies, Santa Clara, CA) and a UniProtKB/Swiss-Prot mouse protein database (16,612 proteins, UniProt.org, release 2013_05). Data files were extracted with the following parameters: fixed modification = carbamidomethylation of cysteine, scans with same precursor mass merged by spectral similarity within tolerances (retention time +/- 10 sec, mass +/-1.4 m/z), precursor charge maximum z = 6, precursor minimum MS1 *S/n* = 10, and ^12^C precursor m/z assigned during extraction. Extracted files were searched with parameters: enzyme = trypsin, species = *Mus musculus*, fixed modification = carbamidomethylation of cysteine, variable modifications = oxidized methionine + pyroglutamic acid + hydroxylation of proline, maximum number of missed cleavages = 2, minimum matched peak intensity = 30%, precursor mass tolerance = 10 ppm, product mass tolerance = 30 ppm, minimum number of detected peaks = 4, maximum precursor charge = 3. Search results were validated at the peptide and protein levels with a global false discovery rate of 1%. Proteins with scores greater than 11.0 were reported, and a list of peptides with scores greater than 6 and scored peak intensities greater than 50% was exported from Spectrum Mill and condensed to a non-redundant peptide formula database using Microsoft Excel. This database, containing peptide elemental composition, mass, and retention time was used to extract MS spectra (M0-M3) from corresponding MS-only acquisition files with the Find-by-Formula algorithm in Mass Hunter Qualitative Analysis software (version B.05.00, Agilent Technologies, Santa Clara, CA). MS spectra were extracted with parameters: extracted ion chromatogram integration by Agile integrator; peak height > 10,000 counts; include spectra with average scans > 12% of peak height; no MS peak spectrum background; unbiased isotope model; isotope peak spacing tolerance = 0.0025 m/z plus 12.0 ppm; mass and retention time matches required; mass match tolerance = +/- 12 ppm; retention time match tolerance = +/- 0.8 min; charge states z = +2 to +4; chromatogram extraction = +/- 12 ppm (symmetric); EIC extraction limit around expected retention time = +/- 0.6 min.

**Stable Isotope LAbeling in Mammals (SILAM) quantitation**

Ratios of light to heavy peptide peak areas were determined using MS spectra extracted from MS-only acquisition files. The peptide formula databases used for peptide isotope analyses were modified to extract light and heavy peptide MS spectra (M0-M2) with the Find-by-Formula algorithm in Mass Hunter Qualitative Analysis software (version B.05.00). MS spectra were extracted with similar parameters to those used for peptide isotope analysis (described in the above “**LC-MS/MS analysis details”** section) except minimum peak height was set to 5,000 counts. SILAM ratios were calculated as the sum of the light peptide M0, M1 and M2 peak areas divided by the sum of the heavy peptide M0, M1 and M2 peak areas. Permissible SILAM ratios were limited to the range 0.02 to 20. Peptide level outliers (2 standard deviations) were removed prior to rolling up SILAM ratios to the protein level.

**Proteomics data calculations: replacement rate constant (*k*), relative pool size (RPS) and within proteome absolute synthesis rates (WPASR) for individual proteins**

Data from individual biological samples were filtered to exclude protein measurements with fewer than two peptide spectra measurements per protein.

### For each protein in each mouse, a replacement rate constant (k, fraction new protein per day) was calculated as:

where *f* is the fraction of protein newly synthesized during the labeling period (as measured by deuterium incorporation) and t is the number of days a given mouse was labeled with heavy water. The relative pool size (RPS) for each protein in a given mouse was calculated as:

where L is the abundance of the light version of a given protein coming from our sample and H is the abundance of the heavy version of that same protein coming from the internal heavy SILAM standard.

For each study, if *k* or RPS data were derived for a given protein for both the control and the experimental group, a mean *k* (or RPS) value was calculated for that protein for each group. Proteins that failed to meet certain criteria were excluded from each study. These criteria included: i) identification of the protein in two or more mice per group, ii) a corresponding mean *f* value of less than 75% (in order to avoid error in kinetic estimates related to values that are close to asymptote, see *Price et al* (Price et al. 2012))*,*  and iii) a coefficient of variation (CV%) for *k* or RPS data of less than 30% for each group.

For those proteins for which mean *k* and RPS data were available in both groups, the within proteome absolute synthesis rates of those proteins were calculated as:

where n represents either the control or experimental group.

**References**

Blackwell BN, Bucci TJ, Hart RW & Turturro A (1995) Longevity, body weight, and neoplasia in ad libitum-fed and diet-restricted C57BL6 mice fed NIH-31 open formula diet. *Toxicol. Pathol.* 23, 570–582.

Bruss MD, Thompson ACS, Aggarwal I, Khambatta CF & Hellerstein MK (2011) The effects of physiological adaptations to calorie restriction on global cell proliferation rates. *Am. J. Physiol. Endocrinol. Metab.* 300, E735–745.

Flurkey K, Papaconstantinou J & Harrison DE (2002) The Snell dwarf mutation Pit1(dw) can increase life span in mice. *Mech. Ageing Dev.* 123, 121–130.

Harrison DE, Strong R, Sharp ZD, Nelson JF, Astle CM, Flurkey K, Nadon NL, Wilkinson JE, Frenkel K, Carter CS, Pahor M, Javors MA, Fernandez E & Miller RA (2009) Rapamycin fed late in life extends lifespan in genetically heterogeneous mice. *Nature* 460, 392–395.

Miller RA, Harrison DE, Astle CM, Baur JA, Boyd AR, de Cabo R, Fernandez E, Flurkey K, Javors MA, Nelson JF, Orihuela CJ, Pletcher S, Sharp ZD, Sinclair D, Starnes JW, Wilkinson JE, Nadon NL & Strong R (2011) Rapamycin, but not resveratrol or simvastatin, extends life span of genetically heterogeneous mice. *J. Gerontol. A. Biol. Sci. Med. Sci.* 66, 191–201.

Miller RA, Harrison DE, Astle CM, Fernandez E, Flurkey K, Han M, Javors MA, Li X, Nadon NL, Nelson JF, Pletcher S, Salmon AB, Sharp ZD, Van Roekel S, Winkleman L & Strong R (2013) Rapamycin-mediated lifespan increase in mice is dose and sex dependent and metabolically distinct from dietary restriction. *Aging Cell*.

Price JC, Khambatta CF, Li KW, Bruss MD, Shankaran M, Dalidd M, Floreani NA, Roberts LS, Turner SM, Holmes WE & Hellerstein MK (2012) The effect of long term calorie restriction on in vivo hepatic proteostatis: a novel combination of dynamic and quantitative proteomics. *Mol. Cell. Proteomics MCP* 11, 1801–1814.
